# The Educative Work of the School Doctor

**Published:** 1915-06-19

**Authors:** 


### THE EDUCATIVE WORK OF THE SCHOOL DOCTOR.

### Practical Points on Lectures to Parents.

The school doctor's work does not merely consist in examining school children and compiling invalidity statistics. This is now generally accepted. It has been recognised that his usefulness extends much farther afield, and that he is an important asset to the authority under which he works, while to the community his value is no less. Nearly all education authorities now demand of their school doctors that these should take some interest in educating the public in questions of hygiene. At the schools themselves the medical inspectors frequently get opportunities to talk to the children and the parents. In this way they can inculcate simple lessons of hygiene, while, if they work harmoniously with the teachers, it is easy enough to arrange to hold equally simple health talks and lectures for both parents and children. This is an important branch of the school doctor's work, for it enables him to supplement his usefulness in indirect ways. Much of the disease he sees at the schools is the result of ignorance. Such defects as static deformities, unsound teeth, tuberculous lesions, defective vision, and defective hearing are the results of things that can be prevented by proper attention in early age to slight ailments, or are the effects of inhygienic methods of life, bad feeding, and insanitary surroundings, which are again the results of ignorance or carelessness. By pointing out these facts in an interesting and simple manner the school doctor can do much to awaken the health sense of the community.

### Lecture Experiences.

It is, therefore, necessary that he should devote some part of his time to this educative work, and that he should every year plot out a simple programme, as diversified as it can possibly be made. Probably he will be able to lecture at the schools, after class hours, where he will have the advantage of a lantern and a good operator. Otherwise he must arrange with some local voluntary body for the loan of a hall or large room for these meetings. The teachers and children will advertise the time and nature of the lectures, but here, again, local enterprise will often come to his assistance. The writer's most successful meetings were engineered by the head teacher, assisted by members of the Care Committee. It is well to give some preliminary thought to the lecture itself. Some medical men think that it is easy enough to talk on health subjects. So it is—in the manner that most medical lecturers use, and that is in vogue among teachers of first-aid and ambulance classes. But it must be remembered that the parents come to the school doctor's lectures not to hear the elements of anatomy and physiology but to get something that is of practical benefit to them. If questions are encouraged at these meetings—and they should be encouraged—for only by questioning can the audience amplify its knowledge and get at the real meaning of obscure points; the lecturer will have to be prepared for all sorts of inquiries. If he does not know the rudiments of domestic economy he will be badly "grounded" by some local peculiarity which he may not have taken into consideration—the price of carrots seriously upset the writer on one occasion when he pointed out to a meeting of parents the advantage of giving their children carrot mash and carrot soup. His Munich experience had taught him that carrots were about the cheapest vegetable that the housewife could invest in, but one of the parents gravely explained that the price of carrots was higher than that of beets or turnips, and unfortunately he had had no practical experience of the value of these two roots as substitutes for his much-advertised carrot *potage*.

Thus it is well for the lecturer to investigate certain points beforehand. Here the advantage of questioning and free discussion is fully shown. Such discussion teaches all who listen, the lecturer as well as the parents. The writer has received many a valuable hint and "tip" from some of his audience at lectures he has given; the frank confession of ignorance on the doctor's part seems to stimulate his audience; they are mildly gratified that they can "tell doctor something he don't know," and they listen to his remarks thereafter with more attention and respect. There is not the slightest fear that such a confession of ignorance will disrate the lecturer in the eyes of his audience. The mother with a big family will take it for granted that even professional men cannot be so wise as she is, and that her experience is far in excess of that of the rest of mankind.

A little attention to the creature comforts at these meetings undoubtedly adds to their success. The hall should be well lighted and comfortably warm; if simple refreshments in the shape of tea and bread and butter are provided, the audience will appreciate them. It may be said that such arrangements are in the nature of bribes, but that is not exactly the way to look at them. Most parents are working people; they must give up a certain amount of their working time in order to be able to attend the lectures, and this fact should be taken into consideration. In one district the parents themselves volunteered to provide refreshments at the afternoon meetings, and both mothers and fathers attended with great and commendable regularity.

### Sidelights on Technicalities.

It is best to make a rule from the start that these lectures are intended for parents, and that no children, not even those in arms, should be allowed to be present. A crying baby disturbs a meeting far more than a rabid anti-vaccinationist does, and you cannot deal with the former as you would with the latter. If you know your subject and keep your temper, the odds are that your audience will be heartily with you in your struggle against the anti-vaccinationist; but you will never get them to regard your fight with the baby as other than an exhibition of professional brutality. Further, at meetings attended solely by adults, by parents, you can deal with matters in a more plain-spoken way than in a mixed audience of juveniles and adults. And the average working-class parent appreciates plain-spoken truths. If you use a little homely slang now and again they will also appreciate it. But they will not appreciate technicalities. A lecturer who talks glibly about the "intestines" may possibly be surprised to find what a construction a South London audience, for instance, places upon his remarks. School doctors who are familiar with the ludicrous interpretations that school children sometimes place upon unfamiliar words will not need to be warned on this point. The writer remembers a schoolboy of average intelligence who sang "The Recessional" with great gusto on Empire Day, and when asked to explain what "reeking tube and iron shard" meant declared that they were "smoking chimbley and the mudguard of a motor-'bus." So use "guts," "lights," "sweet-breads," and similar words by preference. If you cannot make your meaning clear without using hospital expressions, you have no business to lecture at a parents' meeting.

